# P-432. Genotypic Characteristics of Emerging Non-vaccine Serotypes: A Report from Prospective Hospital-based Surveillance for Pediatric Invasive Pneumococcal Diseases in South Korea, 2016-2023

**DOI:** 10.1093/ofid/ofaf695.648

**Published:** 2026-01-11

**Authors:** Dayun Kang, Hyun Mi Kang, Dong Hyun Kim, Nam Hee Kim, Yae-Jean Kim, Yun-Kyung Kim, Chun Soo Kim, Han Wool Kim, Su Eun Park, Eun Song Song, Jong Gyun Ahn, Byung Wook Eun, Joon Kee Lee, Jina Lee, Taekjin Lee, Hyunju Lee, Dae Sun Jo, Eun Young Cho, Hye-Kyung Cho, Jae Hong Choi, Ki Wook Yun, Eun Hwa Choi

**Affiliations:** Department of Pediatrics, Seoul National University Children’s Hospital, Seoul, Seoul-t'ukpyolsi, Republic of Korea; The Catholic University of Korea, Seoul, Seoul-t'ukpyolsi, Republic of Korea; Inha University School of Medicine, Incheon, Inch'on-jikhalsi, Republic of Korea; Inje University Ilsan Paik Hospital, Seoul, Seoul-t'ukpyolsi, Republic of Korea; Samsung Medical Center, Seoul, Korea, Seoul, Seoul-t'ukpyolsi, Republic of Korea; Korea University Ansan Hospital, Seoul, Seoul-t'ukpyolsi, Republic of Korea; Keimyung University School of Medicine, Seoul, Seoul-t'ukpyolsi, Republic of Korea; Hallym University Sacred Heart Hospital, Hallym University College of Medicine, Seoul, Seoul-t'ukpyolsi, Republic of Korea; Pusan National University Children's Hospital, Yangsan si, Kyongsang-namdo, Republic of Korea; Chonnam National University, Seoul, Seoul-t'ukpyolsi, Republic of Korea; Severance Children’s Hospital, Yonsei University College of Medicine, Seoul, Seoul-t'ukpyolsi, Republic of Korea; Eulji University Eulji General Hospital, Seoul, Seoul-t'ukpyolsi, Republic of Korea; Chiungbuk National Hospital, Cheongju, Ch'ungch'ong-namdo, Republic of Korea; Asan medical center, Seoul, Seoul-t'ukpyolsi, Republic of Korea; CHA Bundang Medical Center, Seongnam, Kyonggi-do, Republic of Korea; Seoul National University Bundang Hospital, Seongnam-si, Kyonggi-do, Republic of Korea; Jeonbuk National University Medical School, Seoul, Seoul-t'ukpyolsi, Republic of Korea; Chungnam National University Hospital, Seoul, Seoul-t'ukpyolsi, Republic of Korea; Ewha Womans University Mokdong Hospital, Seoul, Seoul-t'ukpyolsi, Republic of Korea; Jeju National University Hospital, Jeju, Korea, Cheju-do, Republic of Korea; Seoul National University Children's Hospital, Seoul, Seoul-t'ukpyolsi, Republic of Korea; Seoul National University Children's Hospital, Seoul, Seoul-t'ukpyolsi, Republic of Korea

## Abstract

**Background:**

In South Korea, the 10-valent and 13-valent pneumococcal conjugate vaccines (PCVs) have been part of the national immunization program since 2014, and 15-valent was recently introduced in 2024. To understand the current dynamics of pneumococcal serotype distribution in South Korea, we investigated the genotypes of *Streptococcus pneumoniae* isolates from pediatric invasive pneumococcal disease (IPD).

**Figure:**
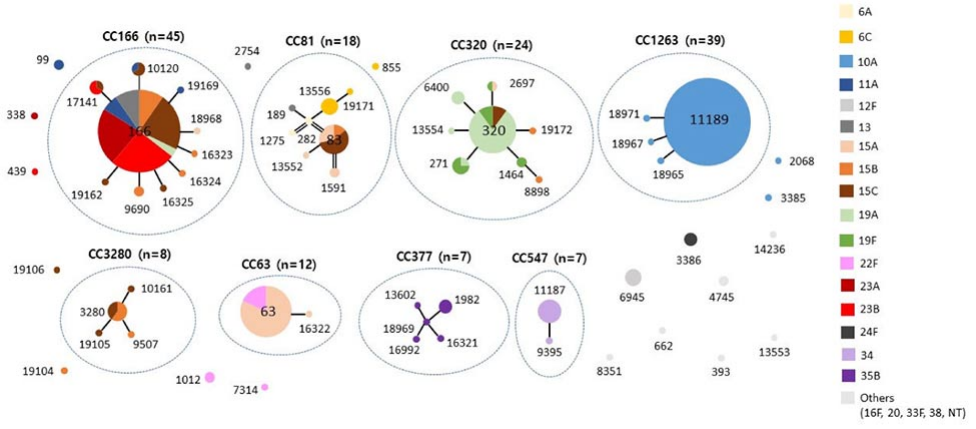
eBURST diagram of multilocus sequence typing from invasive pneumococcal isolates, from 2016 to 2023

**Methods:**

IPD cases from children under 19 years of age were collected through a prospective hospital-based surveillance at 20 hospitals between 2016 and 2023 in South Korea. Serotypes were determined using the Quellung reaction. Genotypes were determined by MLST. Alleles and sequence types were submitted to the web database for assignment. The eBURST diagram was created using the Phyloviz program. Distribution of serotype and genotype was compared between the pre-COVID-19 period (2016-2019) and the during/post-COVID-19 period (2020-2023).

**Results:**

Among the 187 cases with determined serotypes, the most common were 10A (21.9%), 15C (11.8%), 15A (9.1%), 15B (8.0%), 19A (7.5%), and 23B (5.9%). Compared to the pre-COVID-19 period, serotype 23B increased from 0.9% to 14.3% (P < 0.001) and serotype 6C from 0.9% to 7.1% (P = 0.029), while serotype 10A declined from 27.4% to 12.9% (P = 0.018).

The most common clonal complex (CC) was CC166 (24.1%), followed by CC1263 (20.9%), CC320 (12.8%), and CC81 (9.6%). The most prevalent sequence type (ST) was ST11189 (19.3%), followed by ST166 (16.6%). The decrease in serotype 10A was associated with a reduction in the dominant ST11189, while the increases in serotypes 23B and 6C were linked to ST166 and ST13556, respectively, in the during/post-COVID-19 period.

**Conclusion:**

A notable shift in serotype and genotype distribution of pediatric IPD has occurred in South Korea, with the emergence of serotype 23B-CC166 and serotype 6C-CC81. These findings underscore the need to consider emerging non-vaccine serotypes in future immunization strategies to prevent IPD in children.

**Disclosures:**

All Authors: No reported disclosures

